# Association of Gallstone and Polymorphisms of ABCB11 Gene among the Adult Patients in Iran: A Case Control Study: Gallstone and ABCB11 Gene

**DOI:** 10.31661/gmj.v14i.4066

**Published:** 2025-10-28

**Authors:** Sedigheh Yektamoghaddam, Mohammad Mahdi Forghanifard, Vajiheh Zrrinpour, Mojtaba Fathi

**Affiliations:** ^1^ Department of Biology, Da.C., Islamic Azad University, Damghan, Iran; ^2^ Department of Biochemistry and Genetics, School of Medicine, Qazvin University of Medical Sciences, Qazvin, Iran

**Keywords:** Cholelithiasis, ABCB11 Variant, Genetic Variations, Digestive Conditions, Bile Salt Export Pump, BSEP

## Abstract

**Background:**

Background: Gallstone disease (GSD) is a prevalent gastrointestinal condition influenced by genetic, environmental, and dietary factors. The ATP-binding cassette subfamily B member 11 (ABCB11) gene, encoding the bile salt export pump (BSEP), plays a critical role in bile acid transport. Polymorphisms in ABCB11 have been implicated in gallstone pathogenesis, yet evidence from Middle Eastern populations, particularly Iran, remains limited. Our study seeks to address these knowledge gaps by evaluating the association between rs2287622 and hepatic function markers in a distinct population.

**Materials and Methods:**

Materials and Methods: This investigation, conducted in a medical facility with matched participants, involved 100 individuals diagnosed with GSD and 100 comparable healthy volunteers matched by age and gender. We used a targeted PCR method (ARMS-PCR) for variant detection. Laboratory tests evaluated blood fats and hepatic indicators. We applied logistic regression modeling to explore the connection between rs2287622 and GSD, controlling for influencing variables.

**Results:**

Results: The mean age of cases was significantly higher than controls (56.74 ± 16.25 vs. 43.07 ± 14.68 years, P0.001). Cases exhibited elevated serum levels of SGOT (P=0.021), SGPT (P=0.016), ALP (P=0.001), and direct bilirubin (P0.001). No significant differences were observed in allele frequencies (P=0.78) or genotype distributions (P=0.24) of ABCB11 polymorphisms between groups. TT was the most prevalent genotype, with no significant associations found between genotype and clinical parameters or GSD risk.

**Conclusion:**

Conclusion: This study found no significant association between ABCB11 polymorphisms and GSD among Iranian adults. Future research with larger samples and functional analyses is recommended to clarify the genetic determinants of GSD in this population.

## Introduction

Gallstone disease (GSD), also known as cholelithiasis, is a common gastrointestinal disorder characterized by the formation of gallstones in the biliary tract, particularly in the gallbladder [1]. The prevalence of gallstone disease varies significantly across populations, influenced by genetic, environmental, and dietary factors. It has been reported as a major cause of morbidity, imposing substantial healthcare burdens globally [2]. While the global prevalence of gallstones in pregnancy has been estimated at 3.6% based on a systematic review and meta-analysis , prevalence in the general population is reported to range from 10-15% in Western countries and lower in some Asian and Middle Eastern regions [3]. Gallstones can lead to severe complications, including cholecystitis, biliary obstruction, and pancreatitis, necessitating early identification of risk factors for prevention and management [4][5].

Genetic variations in bile salt transporters have been increasingly recognized as important factors influencing hepatic function and susceptibility to cholestatic liver diseases [6]. The ATP-binding cassette subfamily B member 11 (ABCB11) gene encodes the bile salt export pump (BSEP), a crucial transporter responsible for the efflux of bile salts from hepatocytes into the bile canaliculi [7]. Dysregulation of this transporter has been implicated in various hepatobiliary disorders, including intrahepatic cholestasis, gallstone disease, and drug-induced liver injury (DILI) [8].

Among the identified single nucleotide polymorphisms (SNPs) in ABCB11, rs2287622 (V444A) has garnered attention due to its potential role in modulating BSEP function and bile acid metabolism [9][10][11]. However, its precise functional consequences remain controversial and require further investigation.

Previous studies have suggested that the rs2287622 variant may influence bile acid transport efficiency and predispose individuals to cholestatic conditions [12]. Notably, this SNP has been associated with altered hepatic expression of ABCB11 and changes in bile salt homeostasis, potentially contributing to an increased risk of hepatobiliary diseases [13]. Despite these associations, few studies have examined its impact in specific populations or within the context of gene-environment interactions. The influence of rs2287622 on disease susceptibility may be modulated by additional genetic and environmental factors, such as diet, hormonal regulation, and exposure to hepatotoxic agents [14][15].

Given the biological significance of ABCB11 and its role in bile acid metabolism, we hypothesize that the rs2287622 variant may be associated with an increased risk of hepatobiliary dysfunction in our study population. This hypothesis is grounded in prior mechanistic and epidemiological evidence suggesting that genetic alterations in bile salt transporters contribute to interindividual variability in liver disease susceptibility [6]. To address these knowledge gaps, our study aims to (1) assess the association between rs2287622 and hepatic function markers, (2) explore potential interactions with clinical and environmental variables, and (3) provide insights into the genetic basis of bile acid transporter-related disorders. These findings could help elucidate the pathophysiological mechanisms underlying ABCB11-mediated liver dysfunction and inform personalized risk assessment strategies.

### Objectives

Our study seeks to address these knowledge gaps by evaluating the association between rs2287622 and hepatic function markers in a distinct population. Unlike prior research, which has largely focused on Western cohorts, our study examines this genetic variant in a different ethnic group, potentially uncovering population-specific genetic effects. Furthermore, we assess interactions between rs2287622 and key environmental and clinical factors, providing a more comprehensive understanding of its role in hepatobiliary dysfunction. By elucidating the genetic basis of bile acid transporter-related disorders, our findings may contribute to improved risk assessment and personalized strategies for managing gallstone disease and related hepatobiliary conditions.

## Materials and Methods

### Study Design and Population

This hospital-based matched case-control study was conducted at the Research Institute for Gastroenterology and Liver Diseases of Golsar Hospital, Rasht, Iran. A total of 200 participants were enrolled, including 100 patients diagnosed with GSD and 100 matched controls. The control group was matched based on age (within a five-year range) and gender to ensure comparability.

· Inclusion and Exclusion Criteria: Inclusion criteria: Participants aged 18 years or older with a confirmed diagnosis of GSD within the past month.

· Exclusion criteria: Pregnant or breastfeeding women, individuals with a history of intestinal disorders, autoimmune diseases, cancer, or inflammatory/infectious diseases.

Control subjects were selected from hospital departments other than gastroenterology, ensuring they had no history of GSD or other hepatobiliary disorders. Their liver health was confirmed via ultrasound examinations. The rationale for selecting hospital-based controls, rather than community controls, is to minimize potential biases related to healthcare access and lifestyle factors, which could influence the study outcomes [16][17].

The study was conducted in accordance with the Declaration of Helsinki and was approved by the Ethics Committee of Damghan Azad University (IR.IAU.DAMGHAN.REC.1398.006). Written informed consent was obtained from all participants prior to enrollment.

### DNA Isolation and Genotyping

Peripheral venous blood samples were collected in the morning after a 10-12 hour fasting period using EDTA-containing tubes and stored at -20°C until analysis. Genomic DNA was extracted using the Tiangen Genomic DNA Isolation Kit following the manufacturer’s protocol. The purity and concentration of extracted DNA were assessed using spectrophotometry (Pharmacia Biotech GeneQuant II) and visualized via 1% agarose gel electrophoresis.

### Genetic Analysis and ARMS-PCR Technique

Polymorphism data were obtained from the National Center for Biotechnology Information SNP Database. The allele-specific amplification-refractory mutation system-polymerase chain reaction (ARMS-PCR) technique was employed for genotyping the rs2287622 SNP in the ABCB11 gene. The primers for ARMS-PCR were as follows in Table-1.

### PCR Conditions

• Reaction volume: 20 μL

• Thermal cycling conditions:

o Initial denaturation: 95°C for 5 min

o Secondary denaturation: 95°C for 30 sec

o Annealing: 60°C for 30 sec (wild-type) and 65.5°C for 30 sec (mutant)

o Extension: 72°C for 30 sec

o Final extension: 72°C for 7 min

o Total cycles: 35

PCR product integrity was confirmed via electrophoresis on 1% agarose gel stained with ethidium bromide. Band visualization was performed under UV light.

### Biochemical Assessments

Fasting blood samples were obtained at baseline and after eight weeks of follow-up. Serum was separated by centrifugation at 2,000 rpm for 10 min and stored at -80°C. Liver function parameters, including serum glutamic oxaloacetic transaminase (SGOT), serum glutamic pyruvic transaminase (SGPT), alkaline phosphatase (ALP), total and direct bilirubin, and uric acid, were measured using standard enzymatic assays with commercial kits (Pars Azmoon, Tehran, Iran).

### Statistical Analysis

Data were analyzed using SPSS version 25 (SPSS Inc., Chicago, IL, USA). Baseline characteristics were summarized using descriptive statistics (mean ± standard deviation for continuous variables and frequency/percentage for categorical variables). Normality of data was assessed via the Shapiro-Wilk test.

### Comparative Analysis

• Normally distributed variables: Independent t-tests were used.

•Non-normally distributed variables: Mann-Whitney U tests were applied.

• Categorical variables: Chi-square (χ²) tests or Fisher’s exact tests (for low expected frequencies) were performed.

• Multiple comparisons: Benjamini-Hochberg correction was applied to control for false discovery rate (FDR).

### Genetic Association Analysis

• Hardy-Weinberg equilibrium (HWE) was evaluated using the chi-square test.

• Genotype and allele distributions were compared between cases and controls using the chi-square test or Fisher’s exact test when applicable.

• Genetic models (dominant, recessive, additive) were analyzed using logistic regression.

### Multivariate Analysis

To assess the association between ABCB11 gene polymorphisms and gallstone disease risk, logistic regression models were constructed:

• Univariate analysis: Odds ratios (OR) with 95% confidence intervals (CI) were computed for individual risk factors.

• Multivariate logistic regression: Adjusted for potential confounders (age, gender, liver function tests) using backward stepwise selection. Variance Inflation Factor (VIF) was calculated to assess collinearity, and variables with VIF >5 were excluded.

• Bootstrap resampling (1,000 iterations) was used to stabilize confidence intervals and reduce bias.

### Genotype-phenotype Analysis

• The association between genotype and liver function markers was examined using one-way ANOVA (for normally distributed data) or Kruskal-Wallis tests (for non-normally distributed data).

• Post-hoc pairwise comparisons were conducted using Tukey’s test (for normally distributed data) or Dunn’s test (for non-normally distributed data), with Bonferroni adjustment for multiple testing.

A P<0.05 was considered statistically significant, and all tests were two-tailed.

## Result

**Table:** Table1. Primer Sequences for ABCB11 and ARMS-PCR Analysis

**Sequences**	**Product size **
**Wild-type Primer **	174 bp
Forward primer: TCTAAATGACCTCAACATGGT	
Reverse primer: TTTGCACTTTACTGTCCCCA	
**Mutant Primer**	236 bp
Forward primer: TGCCAAAACCTCATCCTTGC	
Reverse primer: TCATTTCCCCTGGTTTAATGG	
	

**ABCB11**; ATP-binding cassette, sub-family B member 11, **ARMS-PCR**; amplification-refractory mutation system polymerase chain reaction, **bp**; base pair.

**Table:** Table2. Demographic and Clinical Characteristics of the Study Population by Groups

**Characteristics**	**Group**	
	**Case (Patients) **	**Control (Healthy) **
Age (years)	56.74 ± 16.25	43.07 ± 14.68	
Gender (Male/Female)	31 / 69	30 / 70	
Genotype (TT/TC/CC)	60 / 35 / 5	60 / 36 / 4	
SGOT (U/L)	19.50 [15.00-29.75]	18.00 [15.00-22.00]	
SGPT (U/L)	20.50 [13.25-38.00]	18.00 [13.00-25.00]	
ALP (U/L)	214.00 [163.25-308.75]	185.50 [145.50-221.00]	
Direct Bilirubin (mg/dL)	0.20 [0.20-0.40]	0.20 [0.20-0.20]	
Total Bilirubin (mg/dL)	0.80 [0.60-1.475]	0.80 [0.70-0.90]	
Blood group (A/AB/B/O)	25 / 4 / 16 / 55	24 / 3 / 15 / 58	


**•** Data presented as Number, Mean±SD, and Median [IQR]. **•** P-values calculated by Chi-square, Independent sample T-test, and Mann–Whitney U test.

**Table:** Table3. Frequency of Normal and Mutant Alleles in the Case and Control Group

Genotype	Case	Control	P
TT	60	60	0.78
TC	35	36	
CC	5	4	

**Table:** Table4. Genotype Distributions among the Participants

		**Gender**		**Total**
		**Male**	**Female**	
	**TT**	35	85	120
**Genotype**	**TC**	21	50	71
	**CC**	5	4	9
**Total**		61	139	200

**Table:** Table5. Characteristics of the Study Population by Genotype Stratified in Two Groups

**Genotype**		**Case (Patients) **				**Control (Healthy) **		
	**TT**	**TC**	**CC**	*P _1_*	**TT**	**TC**	**CC**	*P _2_*
Number	60	35	5	---	60	36	4	---
SGOT (U/L)	26.6±3.1	101.8±39.8	21.6±3.6	0.041	19.3±0.8	18.5±0.8	14.8±1.0	0.272
SGPT (U/L)	33.4±7.5	130.6±42.5	23.6±3.9	0.013	18.5±0.8	18.9±1.2	19.8±5.5	0.904
ALP (U/L)	324.2±64.4	446.3±86.4	173.0±7.4	0.352	186.8±6.4	187.3±9.0	243.0±18.0	0.103
Direct Bilirubin (mg/dL)	0.7±0.2	1.2±0.5	0.4±0.1	0.563	0.18±0.01	0.18±0.01	0.13±0.03	0.086
Total Bilirubin (mg/dL)	1.5±0.3	2.2±0.8	1.1±0.4	0.659	0.80±0.01	0.75±0.02	0.65±0.06	0.025


**•** Data presented as Number, Mean±SEM. **•** P-values calculated by Independent sample T-test.

**Table:** Table6. Logistic Regression Analysis of Factors Associated with Gallstone

Variable	β	S.E.	OR (95% CI)	P-value
Age	-0.053	0.014	0.948 (0.922, 0.976)	< 0.001
Gender (Female)	-0.735	0.514	0.479 (0.175, 1.313)	0.153
Genotype TT	reference			
Genotype TC	0.533	0.447	1.704 (0.709, 4.094)	0.233
Genotype CC	-0.638	1.010	0.528 (0.073, 3.824)	0.528
SGOT	0.044	0.017	1.045 (1.011, 1.080)	0.009
SGPT	-0.045	0.025	0.956 (0.910, 1.003)	0.067
ALP	-0.005	0.004	0.995 (0.988, 1.003)	0.224
Direct Bilirubin	-31.540	6.733	0.000 (0.000, 0.000)	< 0.001
Total Bilirubin	4.629	1.309	102.411 (7.865, 1333.458)	< 0.001
Blood group (A)	reference			
Blood group (AB)	0.451	0.986	1.570 (0.227, 10.833)	0.647
Blood group (B)	0.258	0.663	1.295 (0.353, 4.744)	0.697
Blood group (O)	0.579	0.506	1.784 (0.662, 4.804)	0.252

### Characteristics of the Study Population

A total of 100 patients with gallstone disease (case group) and 100 healthy controls were included in the final analysis. Table-2 summarizes the baseline characteristics of participants. The mean age was comparable between groups (Case: 56.74 ± 16.25 years; Control: 55.07 ± 15.68 years; P=0.76). Gender distribution was also similar (Case: 31% male, Control: 30%; P=0.878).

Regarding liver enzymes, significant differences were observed in serum SGOT (P=0.021), SGPT (P=0.016), and ALP (P=0.001). Additionally, participants in the case group had significantly higher direct bilirubin levels compared to the control group (P<0.001). In term of liver enzymes, we found a significant differences between two groups in term of serum concentration of SGOT (P=0.021), SGPT (P=0.016) and ALP(P=0.001). Moreover, participants in the case group significantly have a higher concentration of direct bilirubin compared the control group (P<0.001). After applying Benjamini-Hochberg correction, significant differences remained in SGOT, SGPT, ALP, and direct bilirubin levels.

### Genotypic and Allelic Frequency Analysis

The distribution of normal and mutant alleles of ABCB11 did not differ significantly between cases and controls (P=0.78, Table-3). The most common genotype in both males and females was TT, whereas CC genotype was the least common.

Table-4 details the genotype distribution among study participants. The most prevalent genotype was TT among women (n=85), while the least common was CC (n=4). Similarly, among men, TT was the most common genotype (n=35). No significant differences in genotype distribution between the case and control groups were found (P=0.24). When stratifying participants into two age groups (<60 years and ≥60 years), TT remained the most common genotype in both groups, with no significant association between age and genotype distribution (P=0.29).

### Clinical Characteristics by Genotype

Table-5 summarizes clinical characteristics by genotype. Among patients with gallstones, SGPT concentrations significantly differed by genotype (P=0.013), as did SGOT levels (P=0.041). In the control group, total bilirubin levels varied significantly across genotypes (P=0.025).

### Association between Different Variables and Gallstone Disease Risk

Logistic regression analysis results are presented in Table-6. A significant inverse association was found between age and gallstone disease risk (OR=0.94, 95%CI: 0.922, 0.976; P<0.001). No significant association was observed between gallstone disease and CC genotype (OR=0.528, 95%CI: 0.073, 3.824; P=0.528) or TC genotype (OR=1.704, 95%CI: 0.709, 4.094; P=0.233). Higher SGOT levels were associated with increased odds of gallstone disease (OR=1.045, 95%CI: 1.011, 1.08; P=0.009), as were higher total bilirubin levels (OR=1.504, 95%CI: 1.014, 1.725; P=0.023). Blood group, gender, ALP, and direct bilirubin were not significantly associated with gallstone disease risk (P>0.05).

## Discussion

In this hospital-based case-control study, we investigated the association between the ABCB11 gene polymorphism rs2287622 and gallstone disease (GSD) in an Iranian adult population. Our findings did not demonstrate a statistically significant relationship between this genetic variant and the risk of GSD. Additionally, our analyses of biochemical parameters among different genotypic groups revealed no substantial differences. These findings suggest that the rs2287622 polymorphism alone may not play a pivotal role in the pathogenesis of GSD within this specific population.

Gallstone disease, or cholelithiasis, remains a prevalent and significant health concern worldwide, often leading to debilitating complications such as acute cholecystitis, biliary obstruction, and pancreatitis [18]. The pathogenesis of gallstones is multifactorial, involving both genetic and environmental factors, with cholesterol supersaturation being a critical step in the formation of cholesterol-based stones [2]. While the genetic basis of gallstone disease has been explored in several populations, the specific role of the ABCB11 gene in gallstone formation, particularly in the Iranian population, has not been thoroughly investigated.

Our study found no significant differences in the allele frequencies or genotype distributions of the ABCB11 gene between patients with gallstones and the control group. This lack of association suggests that polymorphisms in the ABCB11 gene may not play a significant role in the genetic susceptibility to gallstone disease in this Iranian population. These results contrast with findings from studies in other ethnic groups, where ABCB11 polymorphisms have been associated with altered bile acid transport and a higher risk of gallstone disease. Specifically, polymorphisms such as G844A (rs1205571) and T543C (rs2230806) have been implicated in gallstone formation in European, Asian, and North American populations, suggesting a possible link between ABCB11 mutations and impaired bile acid excretion, contributing to bile stasis and stone formation [9][19].

One possible explanation for the discrepancy is the genetic diversity across ethnic groups [20] , as variants associated with gallstone disease in European and Asian populations may not be as prevalent or influential in Iranian individuals. Studies show varying frequencies of ABCB11 polymorphisms by ethnicity [21]. For example, in European populations, mutations in the ABCB11 gene have been strongly associated with gallstones, particularly in patients with cholesterol stones, while studies in African populations have shown weaker or no associations. This variation highlights the need for population-specific genetic studies to better understand the genetic underpinnings of complex diseases like gallstone disease [22].

Additionally, environmental factors such as diet, lifestyle, and geographic location contribute significantly to the risk of gallstone formation [23]. In Iran, dietary patterns are undergoing significant changes, with increased consumption of fatty and high-cholesterol foods, coupled with rising rates of obesity and metabolic syndrome [24]. These factors could influence the formation of gallstones independently of genetic predispositions. For example, a high-fat diet can lead to increased cholesterol secretion into the bile, promoting cholesterol supersaturation and subsequent stone formation. Furthermore, obesity, which is common in the Iranian population, is known to alter bile composition and increase the risk of gallstones. These environmental and lifestyle factors may overshadow the genetic influences of ABCB11 polymorphisms, thereby diluting the potential genetic associations [25][26].

The ABCB11 gene encodes the bile salt export pump (BSEP), a crucial transporter responsible for maintaining bile acid homeostasis. Dysfunction or alterations in BSEP activity may contribute to impaired bile flow and supersaturation, predisposing individuals to cholesterol gallstone formation. Previous studies have suggested that the rs2287622 polymorphism affects the function and expression of BSEP, potentially influencing bile composition and lithogenicity [27]. However, the absence of a significant association in our study indicates that either this specific SNP does not exert a meaningful impact on BSEP function in our population, or that other compensatory mechanisms may counterbalance any potential dysfunction.

It is also possible that the role of ABCB11 genetic variations in GSD development is multifactorial and requires the presence of additional genetic modifiers or environmental triggers. Gene-gene interactions involving other transporters, such as ABCG8 and ABCG5, which are involved in cholesterol efflux, may play a more substantial role in gallstone susceptibility. Furthermore, metabolic factors such as obesity, insulin resistance, and dyslipidemia could mediate the effects of genetic variations on gallstone formation [28].

Despite the lack of association between ABCB11 polymorphisms and gallstone disease, our study found significant associations between clinical variables, particularly liver enzyme levels, and the presence of gallstones. Participants with higher concentrations of serum markers such as SGOT, SGPT, and total bilirubin had significantly higher odds of having gallstones. These findings are consistent with the well-established link between liver dysfunction and gallstone disease [29]. Elevated liver enzymes, particularly SGOT and SGPT, are often indicative of liver inflammation or biliary obstruction, both of which can be caused by gallstones. Additionally, increased bilirubin levels are strongly associated with pigment gallstones, which form as a result of excess unconjugated bilirubin in the bile, often seen in patients with conditions like hemolytic anemia or liver disease [30][31].

These clinical observations reinforce the concept that liver function and bile composition are crucial factors in gallstone formation [32][33]. Although our study did not directly assess other factors such as cholesterol metabolism or bile acid synthesis, elevated liver enzymes and bilirubin levels are important biomarkers in the pathophysiology of gallstones [34]. Previous studies have also shown that patients with elevated liver function tests are at an increased risk of developing gallstones, particularly in the setting of metabolic disturbances such as non-alcoholic fatty liver disease (NAFLD) or diabetes [34][35]. It is possible that in our study, environmental factors such as diet, obesity, and liver health may have had a stronger influence on gallstone formation than genetic factors alone.

One of the strengths of our study is the case-control design with well-matched participants in terms of gender and other baseline characteristics. Moreover, we employed ARMS-PCR, a robust genotyping method, ensuring accuracy in allele discrimination. Despite the relatively small sample size, our study contributes to the growing body of literature assessing genetic predisposition to GSD, particularly in Middle Eastern populations where data remain scarce. Our findings provide valuable insights that can guide future genetic association studies in this region.

Additionally, our study accounted for various biochemical markers, allowing for a more comprehensive evaluation of potential interactions between genetic predisposition and metabolic factors. The inclusion of clinical and biochemical parameters provides a more holistic perspective on disease pathophysiology, though future studies should aim to explore these relationships in greater detail.

Several limitations should be acknowledged. First, the sample size may have been insufficient to detect weak associations, particularly for the CC genotype, which was underrepresented. This limited statistical power calls for replication studies with larger cohorts. Second, while we examined a single SNP, other polymorphisms within the ABCB11 gene and interactions with environmental factors (such as diet and metabolic parameters) were not assessed, potentially limiting the scope of our findings. Third, our study did not include functional analyses such as bile salt profiling or gene expression studies, which could provide mechanistic insights into the role of ABCB11 variants in GSD pathophysiology.

Another critical limitation is the potential for population stratification. Given the genetic diversity within the Iranian population, sub-ethnic variations in allele frequency may have influenced our results. Future studies should incorporate ancestry-informative markers or perform genome-wide association studies (GWAS) to minimize confounding due to population stratification.

Furthermore, our study did not consider lifestyle and dietary factors, which are crucial contributors to GSD risk. As dietary habits play a key role in bile composition and cholesterol metabolism, future research should integrate genetic and environmental data to better understand their combined effects on gallstone formation. Investigating potential gene-diet interactions may help identify at-risk individuals and inform targeted prevention strategies.

### Clinical Implications

Although our study did not establish a significant genetic association, it underscores the complexity of GSD pathogenesis and the necessity of considering multiple genetic and environmental determinants. The findings suggest that reliance on single-SNP analyses may be insufficient for understanding the genetic underpinnings of gallstone disease. Future research should focus on comprehensive genome-wide association studies (GWAS) and functional investigations to elucidate the precise contribution of ABCB11 and other bile salt transporter genes in gallstone formation. Additionally, assessing gene-environment interactions, particularly dietary influences, may yield clinically relevant insights.

From a clinical perspective, understanding genetic susceptibility to GSD could enhance risk stratification and allow for more personalized prevention strategies. Although genetic screening for GSD risk is not yet a standard practice, the identification of high-risk variants in larger, well-powered studies could pave the way for targeted dietary or pharmacological interventions aimed at modulating bile composition and reducing gallstone risk.

## Conclusion

In conclusion, our study found no significant association between the ABCB11 rs2287622 polymorphism and GSD risk in an Iranian population. While this genetic variant alone does not appear to contribute meaningfully to disease susceptibility, further large-scale studies incorporating additional genetic markers and functional analyses are warranted to clarify the role of ABCB11 in gallstone formation. Understanding the genetic underpinnings of GSD will ultimately enhance risk stratification and potential therapeutic interventions.

## Conflict of Interest

The authors have no competing interests relevant to the content of this article to declare.
